# Indication and timing in tricuspid interventions

**DOI:** 10.1007/s12574-025-00705-y

**Published:** 2025-10-01

**Authors:** Atsushi Sugiura, Georg Nickenig

**Affiliations:** 1 Department of Cardiology, Nagoya Heart Center, Sunadabashi 1-1-14, Nagoya, 461-0045 Japan; 2 Department of Internal Medicine II, University Hospital Bonn, Bonn, Germany

## Abstract

Tricuspid regurgitation (TR), previously considered a secondary valvular disorder with limited clinical implications, is now recognized as a progressive and prognostically significant disease. The increasing prevalence due to aging populations and common comorbidities, such as atrial fibrillation and heart failure, has underscored the clinical urgency of addressing TR effectively. Transcatheter tricuspid valve interventions (TTVI) have emerged as valuable therapeutic alternatives, especially for patients at high surgical risk. This review addresses critical clinical questions regarding optimal intervention timing, patient selection, and treatment strategies, focusing particularly on disease progression, right-ventricular (RV) function, and recent clinical evidence. It emphasizes the importance of early identification and monitoring through echocardiographic and laboratory parameters, comprehensive risk stratification including pulmonary hypertension assessment, and the practical use of predictive tools such as TRISCORE. We summarize current guidelines for surgical versus transcatheter interventions and discuss advancements and limitations of transcatheter therapies, particularly transcatheter edge-to-edge repair (TEER) and transcatheter tricuspid valve replacement (TTVR). Ultimately, individualized decision-making based on anatomical considerations, RV function, and comorbidity burden is vital to maximizing therapeutic outcomes.

## Introduction

Tricuspid regurgitation (TR) was regarded as a minor or secondary valvular disorder but is increasingly recognized as a condition with significant clinical implications [1]. However, with aging populations and the rising prevalence of comorbidities, such as atrial fibrillation and heart failure, the clinical burden of TR is becoming more apparent. Importantly, TR is not merely a bystander marker of heart failure but is a direct contributor to poor outcomes, including hospitalization and mortality.

Recent advances in transcatheter tricuspid valve interventions (TTVI) have brought new therapeutic options for patients considered high-risk for surgery. The development of TTVI has renewed interest in optimal timing and patient selection for TR interventions. When should intervention for TR be considered? How should treatment modalities—surgical versus transcatheter—be selected? Which patients would benefit most from the treatment of TR? In this review, we summarize the evolving landscape of TR treatment, with a focus on disease progression, right-ventricular function, and recent evidence supporting transcatheter therapies. We also highlight practical considerations for integrating risk stratification and imaging into clinical decision-making.

## Prognostic significance and disease progression

Multiple large-scale studies have demonstrated the independent prognostic impact of TR [1, 2]. Even mild TR at initial echocardiography is associated with an increased risk of mortality, and the risks increase proportionally with TR severity. In patients with heart failure, moderate or severe TR is associated with a twofold increase in mortality [3]. Importantly, this association remains significant regardless of baseline RV function, atrial fibrillation, mitral regurgitation, left-ventricular ejection fraction (LVEF), or pulmonary artery systolic pressure (PASP), underscoring that TR is not an innocent bystander but independently plays a significant role in outcomes. TR often progresses over time, with atrial fibrillation, age over 60, elevated pulmonary artery pressure, and left atrial enlargement identified as predictors of TR worsening [4]. Other factors associated with TR progression include chronic kidney disease, right-ventricular dilatation, and right atrial dilatation [5]. Given this progressive nature, close surveillance with echocardiography and early identification of at-risk patients is paramount.

## Pathophysiology and classification

TR is classified as primary, secondary (atrial or ventricular), or cardiac implantable electrical device (CIED) lead-related [6]. Primary TR stems from structural abnormalities of the valve leaflets, such as in infective endocarditis or rheumatic disease. Secondary TR accounts for the majority of cases and arises from geometrical distortion of the tricuspid apparatus due to right-ventricular (RV) dilatation or right atrial enlargement. Atrial functional TR is increasingly recognized, particularly in patients with longstanding atrial fibrillation. Ventricular TR is often observed in patients with left-sided heart failure or pulmonary hypertension.

**Fig. 1:**
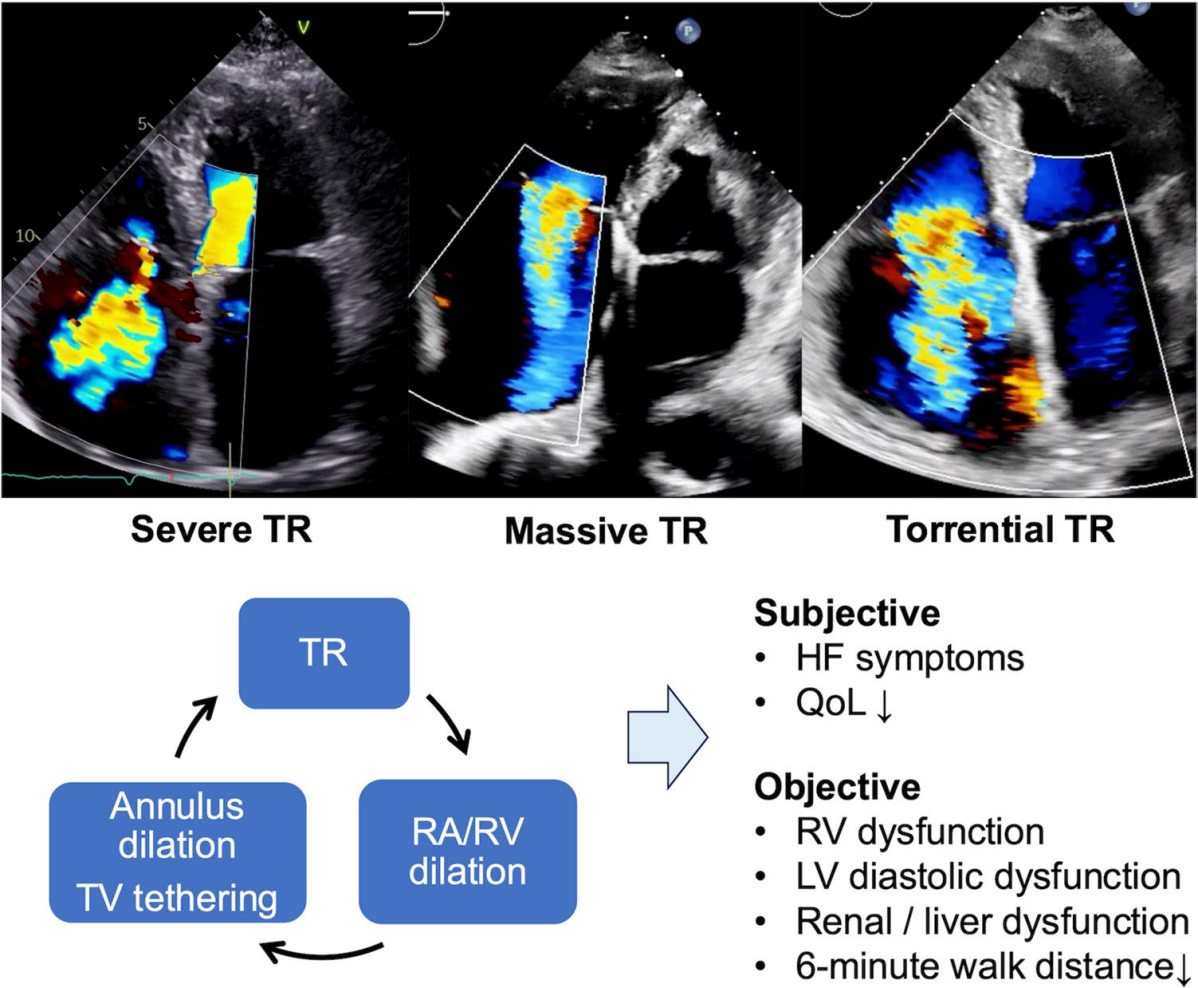
Mechanisms and progression of secondary tricuspid regurgitation. Schematic illustration depicting the pathophysiological mechanisms underlying secondary TR, highlighting the vicious cycle involving RV and annular dilation, leaflet tethering, and progressive valve dysfunction. *RV* right ventricular, *TR* tricuspid regurgitation

Either way, TR initiates a vicious cycle of right heart volume overload, characterized by RV dilation, annular dilation, leaflet tethering, and worsening of TR [7] (Fig. 1). The progression in TR is tightly linked to right heart remodeling. However, TR often eventually leads to clinical deterioration, resulting in the fact that most TR patients present very late in the disease process. Once TR becomes significant, TR in the majority remains significant. It is important to note that even if TR regressed to mild or less, approximately half of the patients develop significant TR again long term [5].

## Right-ventricular function

The assessment of RV function is central to evaluating the timing and feasibility of intervention. Based on guidelines, severe RV dysfunction should be precluded from surgical or interventional therapies [8]. RV dysfunction is associated with increased mortality risk after surgery [9], whereas TEER is safe even in patients with RV dysfunction. However, those patients have poor prognosis even after treating TR [10].

According to the current guidelines, several echocardiographic measurements are recommended (Table 1) [8, 11]. Conventional parameters, such as tricuspid annular plane systolic excursion (TAPSE) and RV fractional area change (RVFAC), offer initial insights but may lack sensitivity in TR patients due to load dependency of the parameters [12]. The combination of TAPSE and RVFAC may be helpful in predicting outcomes [10]. RV longitudinal strain may assist in the early detection of RV dysfunction, given that RV function begins to deteriorate longitudinally first. However, its feasibility for assessing RV function has not been tested in patients with TR. 3D-RV ejection fraction may provide a more accurate estimation of the RV contractility. However, obtaining sufficient quality of 3D images for the tricuspid valve is sometimes challenging. Moreover, the examination and measurement of the 3D-RV ejection fraction is time-consuming.

**Table 1:** Echocardiographic parameters of RV function

TTE measurements	Mild RV dysfunction	Moderate RV dysfunction	Severe RV dysfunction
TAPSE, mm	14–17	10–13	< 10
RVFAC, %	34–37	30–33	< 30
RV TDI S ‘, mm	9–11	6–8	< 6
3D RVEF, %	45–50	35–45	< 35
RV longitudinal strain, %	18–21	14–17	< 14
TAPSE/PASP mm/mmHg	0.41–0.55	0.30–0.40	< 0.30

Summary of echocardiographic criteria used for classifying mild, moderate, and severe RV dysfunction

*RV* Right ventricular, *RVEF* Right-ventricular ejection fraction, *RVFAC* Right-ventricular fractional area change, *TAPSE* Tricuspid annular plane systolic excursion, *TDI* Tissue Doppler imaging, *TTE* Transthoracic echocardiography

Easy, reproducible, and accurate parameters are needed in daily practice. Recent focus has shifted to RV–pulmonary artery (RV–PA) coupling, an integrated measure of RV contractility relative to afterload [13], since the RV is highly sensitive to afterload. This vulnerability contributes to right heart remodeling and exacerbates TR. Conversely, TR itself can lead to volume overload, which may further worsen pulmonary hypertension. Eventually, RV dysfunction may progress to the point that pulmonary hypertension is no longer hemodynamically evident. The RV–PA coupling is commonly estimated noninvasively by the TAPSE/PASP ratio [14]. A coupling of the ratio describes a hemodynamic state where mechanical stroke work is most efficiently transferred to the pulmonary artery circulation. In contrast, uncoupling suggests that RV can no longer maintain forward cardiac output. In patients undergoing TTVI, an echocardiographic TAPSE/PASP ratio < 0.406 is strongly associated with increased mortality and poor procedural outcomes [15]. Although the cutoff needs to be defined in consensus papers, it may be feasible to consider the TAPSE/PASP < 0.30 as severe RV dysfunction and 0.30 ≤ TAPSE/PASP < 0.40 as moderate RV dysfunction [14, 15]. Patients with high preprocedural RV–PA coupling ratios, so-called afterload reserve, have sufficiently preserved ventricular contractile function to tolerate the increased afterload after TR treatment.

## Pulmonary hypertension

The assessment for pulmonary hypertension is a key component of the diagnostic work-up in patients with TR. Despite its importance, PASP can be underestimated in echocardiography. Guidelines recommend a right heart catheterization for evaluating PASP in patients with TR [16]. There are so-called discordant patients who appear not to have pulmonary hypertension but indeed have, based on right heart catheter examination [17]. The RVPA coupling parameter can also be refined by integrating the invasively measured PASP [18]. PASP ≥ 70 mmHg or pulmonary artery resistance ≥ 5 Wood units are deemed as contraindication for TTVI or tricuspid surgery, as the correction of TR might be harmful to the pulmonary circulation [19, 20]. More recent data from the EuroTR registry revealed that PASP ≥ 46 mmHg was the cutoff for predicting mortality and heart failure hospitalization in patients undergoing TTVI [21]. Previous studies showed an inferior outcome of pre-capillary pulmonary hypertension compared to post-capillary pulmonary hypertension [22], where dedicated treatment is indicated. Post-capillary pulmonary hypertension needs a holistic therapeutic approach to reduce left atrial pressure, including optimal medical therapy for heart failure and ablation therapy for atrial fibrillation. Under these circumstances, the benefit of TTVI in patients with pulmonary hypertension needs further investigation. Also, the RV functional reaction to TTVI might be different according to pulmonary hypertension.

## Timing of intervention

One of the most important considerations in managing TR is identifying the “optimal timing” for intervention—when TR is severe enough to justify intervention but before irreversible RV damage occurs. RV dysfunction and multiple comorbidities of patients may limit the potential benefit of TR treatment [23–25].

**Fig. 2:**
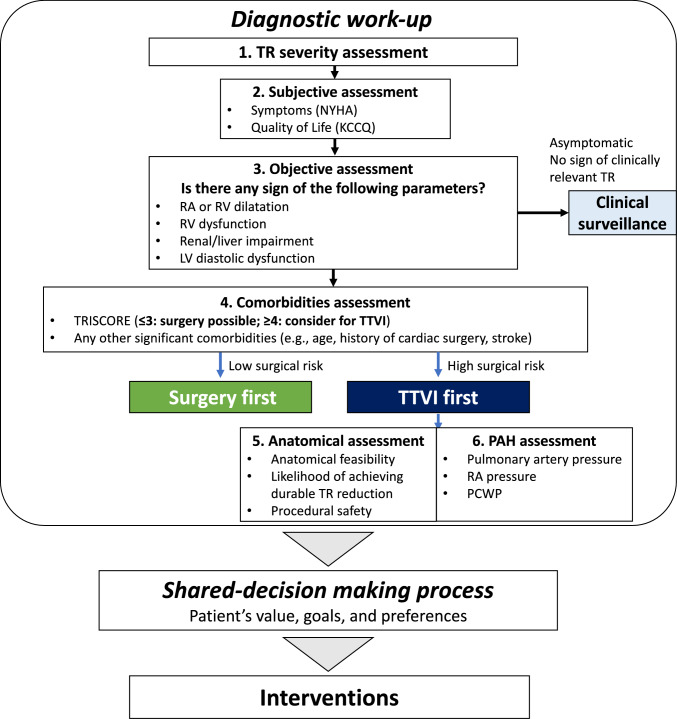
Practical algorithm for determining TR intervention strategy. Flowchart providing clinical decision-making guidance regarding the timing and choice of intervention (surgery versus TTVI) for patients with significant TR, incorporating risk stratification and patient characteristics. *TR* tricuspid regurgitation, *TTVI* transcatheter tricuspid valve treatment

Symptoms, such as leg edema or dyspnea, may be the first sign of hemodynamically significant TR (Fig. 2) [8, 11]. However, in contrast to left-sided valvular disease, TR may progress silently without suffering acute decompensated heart failure; it is not uncommon that the patients are often severely ill with multiple comorbidities by the time they report symptoms. Additionally, some patients may not recognize their symptoms during exercise due to limited physical activity.

Besides symptoms, objective assessments should be added to estimate the disease’s progression, such as echocardiography and laboratory tests (Fig. 2). Dilation of RV and RA, renal and liver function, and LV diastolic function may be surrogates of the disease and can be monitored noninvasively by applying echocardiography. RV dilation is the first structural change if the TR is significant [7]. Renal and liver dysfunction, as assessed by estimated glomerular filtration ratio or total bilirubin, may reflect impaired perfusion and right heart congestion. In contrast, the prognostic value of brain-natriuretic peptide in patients with TR needs to be clarified. LV diastolic dysfunction may be observed in the presence of significant TR [26, 27], since the increased RV pressure may interact and limit the septal wall shift toward the right side during the diastolic phase. Assessing the factors associated with LV diastolic function, such as e’, E/e’, and LA volume index, may be indicative of the hemodynamic significance of TR. Worsening of the aforementioned factors should facilitate timely intervention and not unintentionally postpone the timing of the treatment.

Optimal medical therapy is limited mostly to diuretics and medications targeting the left-side heart. Although diuretics for reducing volume load or rhythm control therapy for reversing remodeling of the right atrium and tricuspid annulus may reduce TR and improve symptoms, neither of these therapies has been demonstrated to improve clinical outcomes in patients with TR. The potential effects of contemporary heart failure medications, such as SGLT2 inhibitors, on the progression of TR and right heart remodeling are still to be investigated. Patients with persistent or worsening signs of right heart congestion despite medical treatment should be referred for tricuspid valve surgery or TTVI.

## Surgical indications

According to current American and European guidelines, surgery for TR is indicated primarily in patients with severe TR undergoing left-sided valve surgery. Isolated TR was traditionally conservatively managed with diuretic therapies. However, such an approach may overlook opportunities for earlier intervention in isolated TR, where progressive RV dysfunction may occur silently. Currently, in patients presenting with symptomatic right heart failure and evidence of RV dilatation, isolated surgical correction or transcatheter therapy may be considered earlier. Holistic assessment of TR patients by institutional heart teams should be employed to guide the referral or therapeutic timing. TRISCORE is a simple risk prediction model of periprocedural mortality after isolated tricuspid valve surgery. The score is calculated based on the following factors: age ≥ 70 years, NYHA functional class III or IV, right-sided heart failure signs, a daily dose of furosemide ≥ 125 mg, glomerular filtration rate < 30 ml/min, elevated total bilirubin, LV ejection fraction < 60%, and moderate or severe RV dysfunction. The risk for mortality increases along with the score.

Although further investigations should be conducted, one may suggest that the cutoff for undergoing surgery is TRISCORE three points or less (Fig. 2). With the low score category, surgery may improve the survival of patients with TR, in comparison to conservative management. Importantly, the benefit of surgery declines as TRISCORE increases (i.e., TRISCORE ≥ 4) [23]. Surgical replacement in intermediate-risk patients or any tricuspid valve surgery in high-risk patients may be harmful due to increased periprocedural mortality.

## Advances in transcatheter therapies and its indication

TTVI should be considered for treating TR in patients at high surgical risk. Given the known high periprocedural mortality in isolated tricuspid valve surgery, patients with intermediate or greater TRISCORE (i.e., ≥ 4 points), elderly patients, or those with multiple comorbidities may be referred to the transcatheter therapies (Fig. 2). Transcatheter edge-to-edge repair (TEER) with the TriClip and PASCAL systems is the safest alternative, demonstrating low periprocedural mortality, significant reductions in TR severity, improvements in symptoms, and enhanced Quality of life for patients [28, 29] **(**Table 2**)**. The safety profile is consistent, irrespective of RV function. Even in patients with severe RV dysfunction at baseline, acute RV deterioration is not seen or, at least, clinically not a problem for short-term outcomes [10]. Nonetheless, anatomical complexity makes achieving adequate TR reduction challenging [27]. The GLIDE score, proposed by Gerçek et al., is a clinical scoring system for predicting TR reduction after tricuspid TEER [30].

**Table 2:** Comparison of TEER and TTVR in current practice

	TEER	TTVR
Anatomical feasibility	Limited by coaptation gap, TR jet location, leaflet configuration	Limited by annular/RV size
Residual TR rate at 30 days	Moderate (residual TR common)	Low (near-complete elimination)
Procedural mortality	Low [25, 26]	Moderate [18, 20, 30, 31]
RV afterload sensitivity	Low	High (afterload mismatch possible)
Pacing requirement	None	10–15% (> 20% in patients without pacemaker at baseline)
Durability (TR ≤ moderate at 1 year)	Good, but residual TR frequent	Excellent

Comprehensive comparison highlighting anatomical feasibility, procedural outcomes, residual TR rates, procedural mortality, RV sensitivity to afterload changes, pacing requirements, and long-term durability between TEER and TTVR approaches

*RV* Right ventricular, *TEER* Transcatheter edge-to-edge repair, *TR* Tricuspid regurgitation, *TTVR* Transcatheter tricuspid valve replacement

Transcatheter tricuspid valve replacement (TTVR) is the other alternative, especially in patients with unfavorable anatomy for TEER, offering substantial TR reduction (Table 2). The TRISCEND pivotal trial, comparing TTVR versus medical therapy alone, demonstrated a substantial reduction in TR, with residual TR ≤ mild at 1 year of 94.1% [20]. The TR reduction was durable until a 1-year follow-up (TR ≤ mild: 95.3%), showing significant reverse remodeling of RV. However, the safety issues remain to be solved. Periprocedural mortality is slightly higher than TEER. RV function might acutely deteriorate after tricuspid valve replacement, since the RV is highly sensitive to changes in afterload. Conduction disturbance may occur in approximately 10–15% of patients. Moreover, more than half of the screened patients declined to undergo the procedure due to the too large or too small anatomy of the tricuspid valve annulus or the right ventricle [31].

In the current clinical practice, TEER remains a working-horse transcatheter device for treating TR in patients with high risk for surgery. For anatomically challenging cases or those unsuitable for TEER, transcatheter TTVR offers a more definitive solution, often achieving near-complete elimination of TR. A scoring system for predicting the feasibility of TEER is yet to be developed to establish a pragmatic device selection for treating TR.

## Appropriate patient selection

One of the most significant unsolved questions is which subjects would benefit the most from the TR intervention. Despite the collectively shown benefit on QoL, symptoms, and heart failure hospitalization, no study has yet shown the survival benefit of TR intervention over medical therapy alone [19, 20, 33]. From a procedural perspective, both transcatheter and surgical TR interventions must achieve the minimal periprocedural mortality and deliver effective, durable TR reduction. The TRILUMINATE pivotal trial highlighted a positive correlation between the degree of TR reduction and the improvement in KCCQ score [19]. Furthermore, a greater increase in the KCCQ score may be associated with improved survival after TEER [33]. TTVR generally achieves more pronounced TR reduction than TEER; however, its procedural safety profile requires further optimization [20]. Surgical approaches to TR are often associated with an increased risk of in-hospital mortality [34]. Therefore, careful patient selection remains paramount to balancing the potential benefits of intervention with the associated procedural risks.

From a patient characteristic perspective, RV function and the burden of comorbidities in patients may affect the benefit of TR treatment; RV function may also influence the prognostic relevance of TR [24]. In the TRISCEND pivotal trial, patients with RVFAC ≥ 35% showed a significantly greater increase in the KCCQ score compared to those with RVFAC < 35%. Similarly, in a single-center cohort study, the outcome benefit from TR reduction was greater in patients with RV ejection fraction ≥ 45% compared to those with RV ejection fraction < 45% [25]. A multicenter study using a propensity score matching analysis suggested that the benefit of TR treatment over medical therapy was pronounced in patients with intermediate RV function but not in patients with severe RV dysfunction or preserved RV function [35]. Similar to the RV function, the burden of comorbidities may also affect the prognostic benefit of TR treatments. A multicenter analysis showed that there was no survival difference between TR therapy and medical therapy in patients with TRISCORE ≥ 6 [23]. These findings underscore the importance that TR should be considered for interventions before patients have severe RV dysfunction or increased burden of comorbidities in which TR therapy might be palliative.

**Fig. 3:**
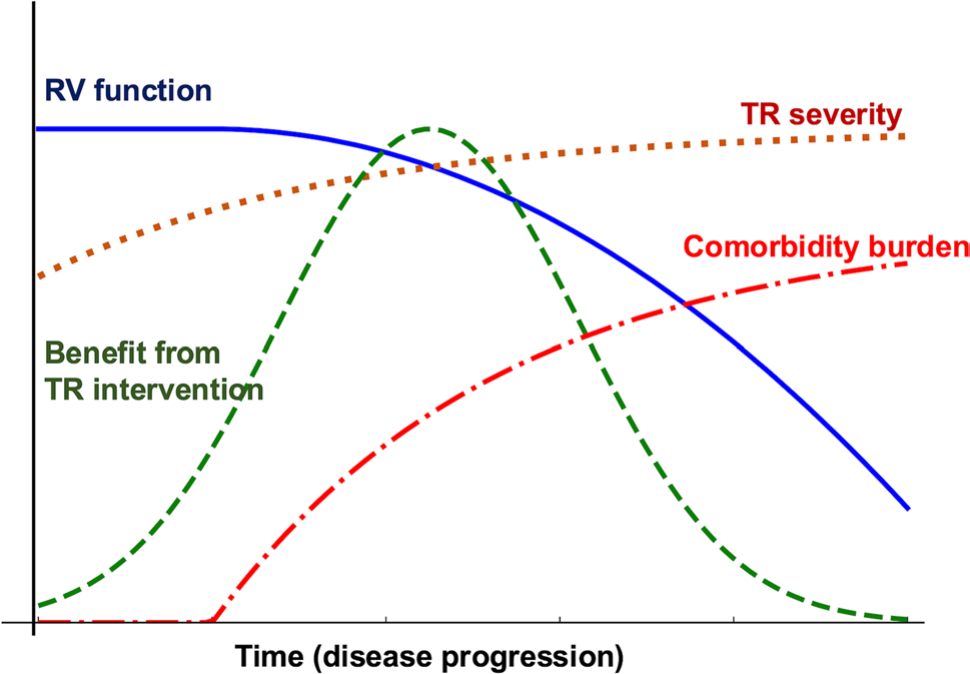
Timing of TR intervention based on RV function, TR severity, comorbidities, and potential benefit from TR treatment. This conceptual diagram illustrates the optimal timing for TR intervention by integrating TR severity, RV function (e.g., TAPSE, RVFAC, TAPSE/PASP), and the burden of comorbidities (e.g., renal/liver dysfunction, advanced age, frailty). The figure emphasizes that the therapeutic benefit is maximized when TR is treated before the development of severe RV dysfunction or multiorgan compromise. Patients with preserved or moderately impaired RV function and manageable comorbidity profiles are most likely to benefit from surgery or transcatheter therapies. Conversely, in patients with advanced disease and multiple comorbidities, interventions may be palliative. Nonetheless, symptomatic relief and risk reduction in heart failure rehospitalization are clinically meaningful outcomes for patients, even in the absence of a proven survival benefit. Therefore, a multidisciplinary institutional heart team should carefully evaluate the potential benefits and risks of each intervention option. *PASP* pulmonary artery systolic pressure, *RVFAC* right-ventricular fractional area change, *TAPSE* tricuspid annular plane systolic excursion, *TR* tricuspid regurgitation

Therefore, to target a meaningful survival benefit, two essential conditions should be met:

1. The procedure must be performed with minimal periprocedural mortality and a high probability of achieving sufficient, durable TR reduction.
2. Patients with severe RV dysfunction or multiorgan failure should be excluded from intervention candidacy (Fig. 3).

## Shared decision-making process for TR interventions

That said, it is essential to acknowledge that prior studies have collectively shown the positive benefits of TTVI on QoL improvement in patients with TR. Furthermore, symptomatic relief and risk reduction in heart failure rehospitalization are clinically meaningful outcomes for patients, even in the absence of a proven survival benefit.

Therefore, a multidisciplinary institutional heart team should carefully evaluate the potential benefits and risks of each intervention option. This assessment should incorporate all relevant clinical parameters in a comprehensive diagnostic work-up. Crucially, this information must be communicated to the patient through a structured shared decision-making process, which integrates the patient’s values, goals, and preferences into treatment planning (Fig. 2). Such a patient-centered approach represents the foundation of modern medical care in the management of TR.

## Conclusions

TR is no longer a benign or secondary finding but a progressive disease with a clear prognostic impact. Indication and timing of TR intervention should be individualized by a multidisciplinary institutional heart team based on a holistic approach. Besides symptoms, integrating objective parameters of RV function and LV diastolic function, as well as renal or liver function, can guide clinical decision-making. Patients with low risk for surgery can safely undergo surgical collection of TR. Transcatheter therapies, TEER and TTVR, have expanded treatment options for high-risk patients, consistently demonstrating QoL improvement and symptomatic relief. For targeting survival benefit, timely intervention—especially before RV dysfunction and multiorgan damage become profound—may be essential. The selection of devices should be based on procedural safety, anatomical feasibility, and the likelihood of achieving durable TR reduction to maximize therapeutic benefit.

## Data Availability

The data that support the findings of this study are available from the corresponding author upon reasonable request.
